# Using a simulation model to assess the significance of temperature and host availability for population dynamics of *Drosophila suzukii* (Diptera: Drosophilidae)

**DOI:** 10.1371/journal.pone.0351723

**Published:** 2026-07-15

**Authors:** John B. McCulloch, Joshua A. Grant, Ye Chu, Ashfaq A. Sial

**Affiliations:** 1 Department of Entomology, University of Georgia, Athens, Georgia, Unites States of America; 2 University of Georgia Cooperative Extension: Barrow County, Winder, Georgia, Unites States of America; 3 Department of Horticulture, University of Georgia Tifton Campus, Tifton, Georgia, Unites States of America; Government College University Faisalabad, PAKISTAN

## Abstract

Spotted-wing drosophila, *Drosophila suzukii* Matsumura (Diptera: Drosophilidae) (SWD), is an important pest of soft-skinned fruit around the globe because of its propensity to oviposit into ripe fruit, directly impacting the marketable portion of a crop. Temperature and host availability are two major factors that influence SWD population dynamics, but predictive tools are absent for blueberries in southern Georgia, USA, where multiple non-crop hosts occur in habitat adjacent to blueberry farms and where high summer temperatures present challenges for SWD population growth. We used temperature and crop and non-crop host phenology in southern Georgia, USA, to drive a physiological age-structure simulation to assess the importance of these factors for SWD. We used the Kolmogorov-Smirnov (KS) test to assess how different simulation conditions compared to trap catch data from 2015–2017. The standard simulation, in which crop host availability peaked in late spring and non-crop host availability peaked in autumn and winter, predicted the decline in trap catches in early summer but did not predict the increase in trap catches during the autumn and winter. High summer temperatures and reduced or declining host availability constrained SWD reproduction, as indicated by increased proportions of adults and decreased proportions of immatures in the simulated population during these periods. The KS test showed that simulation conditions with only non-crop hosts available to SWD matched the trap data the best, compared to the standard simulation with crop and non-crop hosts. Further research to describe adaptations of SWD populations to local conditions and the importance of non-crop hosts for sustaining SWD populations is necessary to improve our understanding of SWD. Improving our knowledge of SWD in these regards will increase the accuracy of predictive tools and advance sustainable management of this worldwide pest.

## Introduction

As poikilotherms, temperature is a key component in the physiology and development of insects [1]. The developmental rate of insects can often be characterized between minimum and maximum temperature thresholds as a near-linear positive relationship, and researchers and farmers use this relationship to measure physiological time (i.e., accumulated degree days) and predict important events in pest and crop phenology. Physiological time is a useful context with which to construct life tables for insects and can improve predictions of life-history events compared to calendar based predictions [2,3]. Wiman et al. (2016) described the relationship between accumulated degree days and the survival and fecundity of spotted-wing drosophila, *Drosophila suzukii* Matsumura (Diptera: Drosophilidae) (SWD) for use in modified Leslie-Matrix population simulations.

Spotted-wing drosophila is a polyphagous pest of soft-skinned fruits, and in extreme cases can cause up to 37% reduced revenue [4,5]. Originating in southeast Asia, SWD was detected in Hawaii, USA, in the 1980s and became established in the continental USA and Europe ca. 2008; currently this insect is present worldwide where suitable hosts are available and climate is favorable for sustained populations [5–10]. Spotted-wing drosophila has multiple generations per year and overwinters as adults that have increased tolerance to colder temperatures and lower relative humidity [11–13]. Current management strategies often rely on conventional insecticides such as organophosphates and pyrethroids, while organic insecticide options are fairly limited and include spinosyn-based products [6,14]. There is an increasing interest in biological control of SWD, including the use of parasitoid, nematode, and microbial agents, as well as cultural control such as exclusion netting [4,6,15,16]. The range of SWD includes different climatic regions, therefore simulation tools are needed to predict SWD population dynamics and inform management options at a regional scale [17–21].

Life history and developmental data have been generated for laboratory populations of SWD originating in different states of the USA (e.g., California, Georgia, Oregon) and from several different countries across the globe (e.g., France, Germany, Italy, Spain) [22–27]. Likewise, development and population simulations have been parameterized with local temperature data for multiple locations in the USA (e.g., California, Oregon, North Carolina) and across the globe (e.g., France, Germany, Italy, Spain) [17–19,21,24,25,27]. Temperature and host availability have been identified as two of the most important factors to predict SWD population dynamics [19,21,27]. The majority of blueberries in Georgia, USA, are grown in the southern part of the state, where summer temperatures regularly exceed 35°C, and where non-crop hosts are present in the landscape surrounding blueberry fields [7,28]. However, there are no predictive tools for SWD populations in southern Georgia, USA. Therefore, simulation tools, currently unavailable, are necessary to predict SWD population dynamics and inform management decisions in this region where high summer temperatures present challenges to SWD population growth [23,29].

This computer simulation study fills a management tools gap for blueberry growers in Georgia, USA. We use functions for SWD survival and fecundity driven by physiological age with temperature data from Alma, Georgia, USA, and crop and non-crop host phenology in southern Georgia, USA, to simulate SWD population dynamics. This simulation was used to assess the impact of host availability and summer temperatures on SWD populations. Simulation results are compared to trap catch data collected at seven sites in southern Georgia, USA. The results of this study improve our understanding of factors influencing SWD population dynamics in a region where non-crop hosts are available to SWD and where high summer temperatures present challenges to SWD survival and reproduction. This additional knowledge will aid blueberry growers in making management decisions for SWD, aiming to improve efficiency and profitability, and reduce the risk associated with pesticide applications for agricultural workers and the environment.

## Materials and methods

### Data collection and uses

Seven southern high-bush and two rabbiteye blueberry varieties were assessed for fruit development (i.e., green fruit, 25%, 50%, 75%, and 95% blue fruit) at the University of Georgia Alapaha Blueberry Research Farm near Alapaha, Georgia, USA. Plots consisted of five plants and were sampled every two weeks from January to June in 2023 and 2024, with a single rating value assigned to the whole plot at each sampling date. These data were used to estimate duration of fruiting (green fruit to 95% blue fruit) for southern highbush and rabbiteye blueberries in southern Georgia, USA. Temperature data from the Georgia Weather Network (University of Georgia) at Alapaha, GA, USA, were used to estimate average growing degree days (base 10°C: 50°F) to peak fruit set (50% blue fruit). These data were used to parameterize Gaussian distributions that described the availability of crop hosts throughout the season for both southern highbush, *Vaccinium corymbosum*, and rabbiteye, *Vaccinium virgatum*, blueberry species. For each blueberry species, the height of the distribution was set to 1 (*a = 1*), was centered on the number of degree days to peak fruit set (*µ = degree days to 50% blue fruit*), and the distribution was assigned a standard deviation equal to one fourth the time from green fruit to 95% blue fruit (*σ = ¼ time from green fruit to 95% blue*). The distribution for each blueberry species (southern highbush and rabbiteye) were added together to describe crop host availability and the sum was adjusted using a scaling factor set to 1 unless otherwise noted.

Data for the occurrence of fruiting potential non-crop host species adjacent to blueberry fields (10m into non-field habitat) were collected every other week from January to May and August to December, and weekly from May to August during 2015 in six counties in southern Georgia: Appling, Bacon, Brantley, Clinch, Pierce, and Ware counties [7]. All potential species were included as non-crop hosts regardless of the suitableness of the host for SWD development [7]. Similar to the use of SWD host phenology data in Italy by Pfab et al. (2018), the number of fruiting non-crop host species per month was used to fit a composite function to describe the proportion of non-crop host species availability throughout the year [8,19]. The PROC REG statement was used to verify that the function fit the observed data (SAS 9.4; SAS Institute; Cary, North Carolina, USA). Furthermore, the scaling factor for non-crop host availability was set to 0.3 unless otherwise noted.

Trap catch data were collected in and around seven blueberry farms in southern Georgia (10m into the crop field, in the border between the crop field and the surrounding unmanaged habitat, and 10m into the unmanaged habitat). Data were collected every two to 21 days from March 11, 2015 to October 4, 2017 and were standardized to the number of adult SWD caught per trap per week. The mean number of SWD per trap per week at each collection date was used to assess the results of each simulation.

### Simulation description

**Fig 1:**
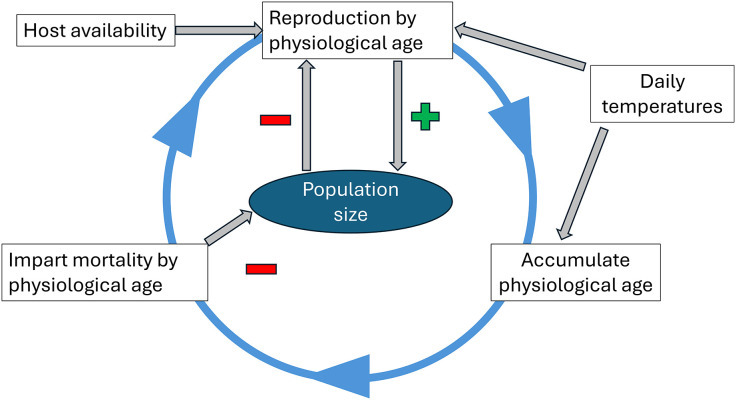
Visual model of the simulation. The simulation started with an initial population. Host availability affects reproduction, lowering the realized fecundity when hosts are less available. Population size also regulates fecundity in a density-dependent manner. Additionally, fecundity is maximized at an optimal reproductive temperature, and departure from that optimum temperature reduces realized fecundity. At the beginning of a time step, cohorts of reproductive age produce eggs according to their physiological age. A census is taken, and then degree days are accumulated for the time step, which ages the cohorts in the population. Mortality is then imparted according to physiological age, and the time step ends. The simulation proceeds with the next time step.

An age-structured Leslie Matrix-style simulation was used to simulate the population dynamics of SWD using temperature and host availability data from southern Georgia, USA. The simulation was initiated with a founding population of ten cohorts of adult SWD, with 500 adults per cohort spaced evenly across the physiological age range for adults [20]. The theoretical fecundity for each cohort was based on physiological age and described by a Cauchy function that approximated the fecundity equation in Wiman et al. (2016). Realized fecundity was calculated for each cohort of reproductive age at each time step by modifying theoretical fecundity with factors describing temperature related fecundity rates, host availability, and the population density of immature SWD: *F*_*real*_ *= F*_*th*_ *× T*_*fec*_ *× H × DD*, where *F*_*real*_ = realized fecundity, *F*_*th*_ *=* theoretical fecundity*, T*_*fec*_ = temperature based fecundity factor*, H* = host availability factor*,* and *DD* = density-dependent fecundity factor [19,26,30]. Fecundity was only allowed within lower and upper reproductive temperature limits unless otherwise noted [20]. The number of eggs produced by females in all cohorts of reproductive age at each time step were summed and constituted a new cohort with physiological age = 0. A census of the population was taken after reproduction at each time step but before the population aged. Cohorts in the population aged according to the degree days accumulated during each time step. Survival of individuals in a cohort was determined by physiological age using a previously described Gompertz function [20]. Degree days were only accumulated above a lower temperature threshold; above an upper temperature threshold, degree days were accumulated at a constant value. Temperature was assumed to fluctuate during each time step (i.e., one day) according to a sine wave parameterized by the minimum and maximum temperatures of that step, and degree days were calculated as the sum of degree-hours at 24 evenly spaced increments across the sine wave [31]. Several important assumptions were that the sex ratio of the population was 1:1 male:female, each time step was one day, and there was no immigration to the population or emigration to other populations. Individuals during the summer and winter were assumed to be sufficiently adapted to those conditions such that extreme temperatures did not affect survival beyond the indirect impact via physiological aging from accumulated degree days [19]. The simulation was initiated one year before the trap catch data to allow a ‘burn-in’ period and reduce the impact of the arbitrary starting population size and age on the output during the time of the trap catch data. The simulation was constructed using Python 3.12.7 in the Spyder development environment, v5. See Fig 1 for a visual representation of the simulation. Local sensitivity analyses were performed for initial population size and age, carrying capacity, proportions of crop and non-crop hosts in the landscape, and for crop host phenology (S1 Fig–S4 Fig). Simulation code and input data can be accessed in the ‘SWD-population-dynamics’ repository on GitHub.

### Modifications to theoretical fecundity

Temperature modifications to theoretical fecundity were validated using data from Tochen et al. (2014): eggs laid per female per day on blueberry at five different constant temperatures were fit with a Gaussian distribution with amplitude *a* = 19.8, center ‘*µ*,’ and sigma ‘*σ*’ using the least squares method. Ranges of values explored for *µ* and *σ* were 20.0–22.6 and 4–6.2 with increments of 0.2 and 0.1, respectively. The PROC REG statement was used to test the fit of the function to the data. For use in the model, the amplitude of the Gaussian function for temperature related fecundity was reduced to 1, which served as a proportion driven by daily temperature with which to modify the theoretical fecundity of SWD females. The host availability factor modified theoretical fecundity as a Holling Type 2 function, which included the amount of resources available and an ‘attack rate’ for the oviposition of SWD onto hosts [19]. The density-dependent fecundity factor modified theoretical fecundity as a function of unutilized resources, given the number of immature SWD in the population and a carrying capacity for available resources [30].

### Simulation modifications

**Fig 2:**
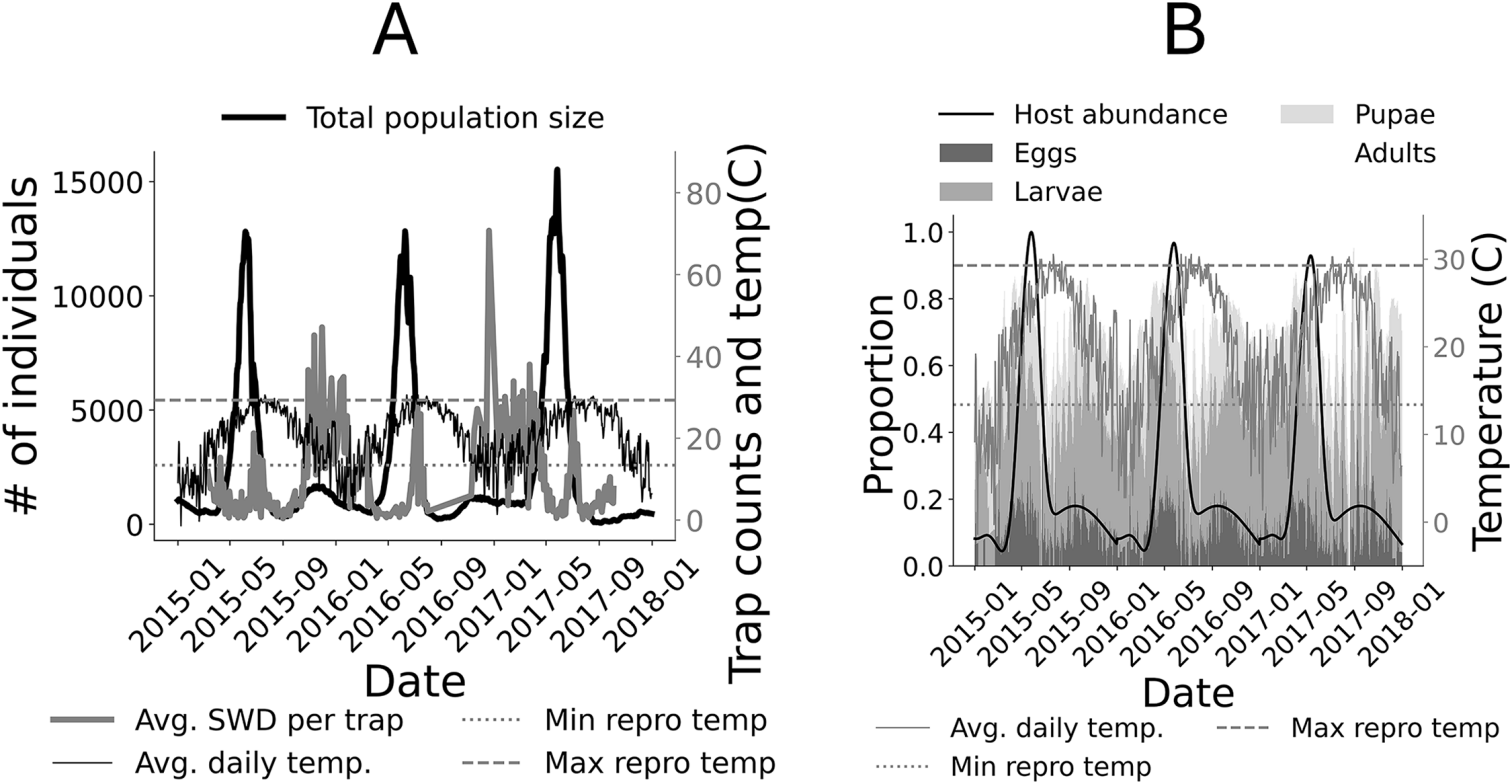
Standard simulation. Temperature and host modifications to fecundity are both implemented in the standard simulation. A) Population density compared to trap catch data, 2015-2017. Simulated population size on left y-axis; trap counts and temperature on right y-axis. B) Proportion of life stages in the simulated population during the period of trap catch data; cumulative proportion of the population for each life stage and host availability on left y-axis; temperature on right y-axis. ‘Repro’ = reproductive; ‘temp’ = temperature.

**Fig 3:**
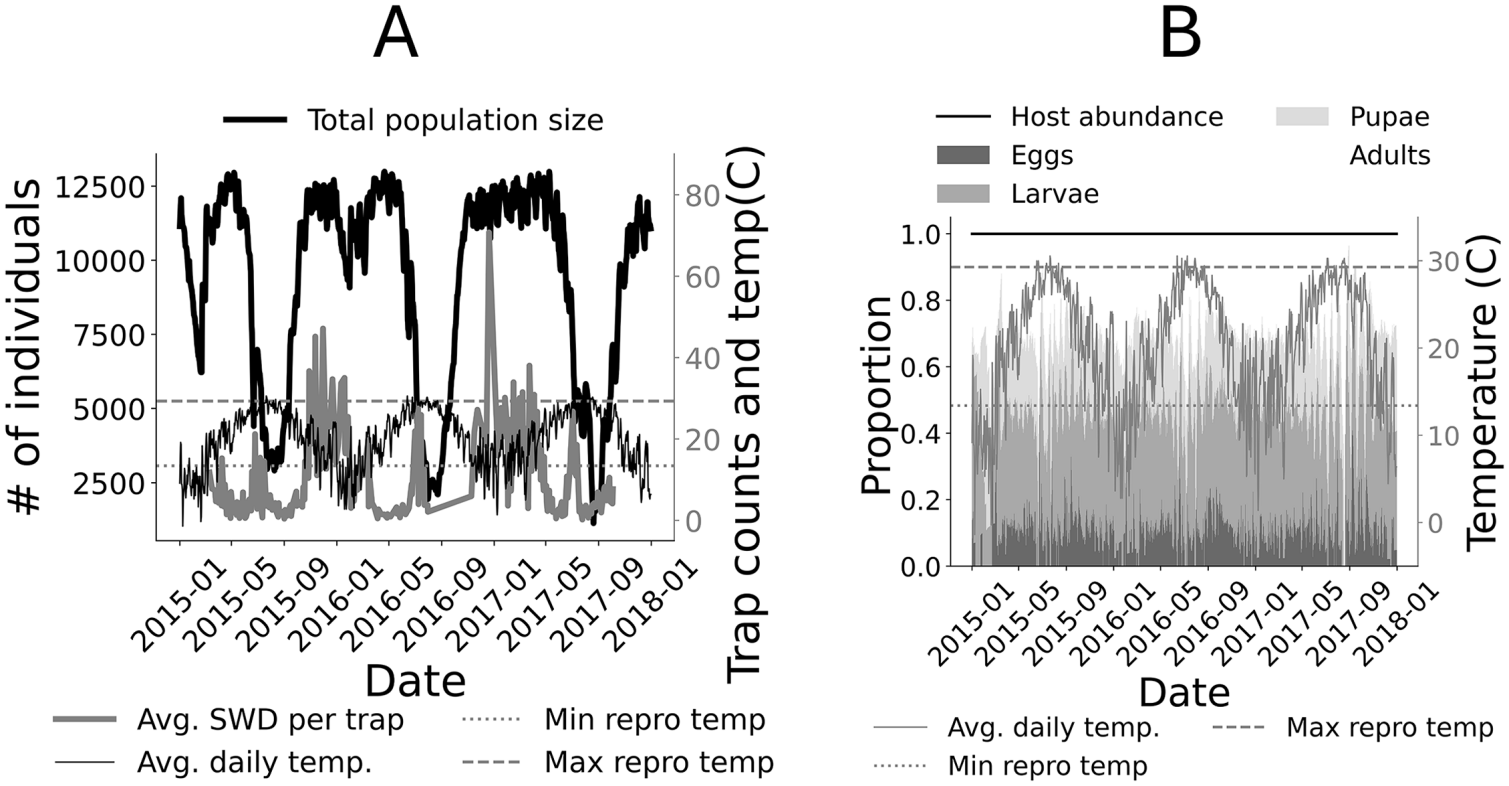
Testing the effect of temperature. Fecundity modifications by host availability were removed to assess the impact of temperature on population dynamics. A) Population density compared to trap catch data, 2015-2017. Simulated population size on left y-axis; trap counts and temperature on right y-axis. B) Proportion of life stages in the simulated population during the period of trap catch data; cumulative proportion of the population for each life stage and host availability on left y-axis; temperature on right y-axis. ‘Repro’ = reproductive; ‘temp’ = temperature.

**Fig 4:**
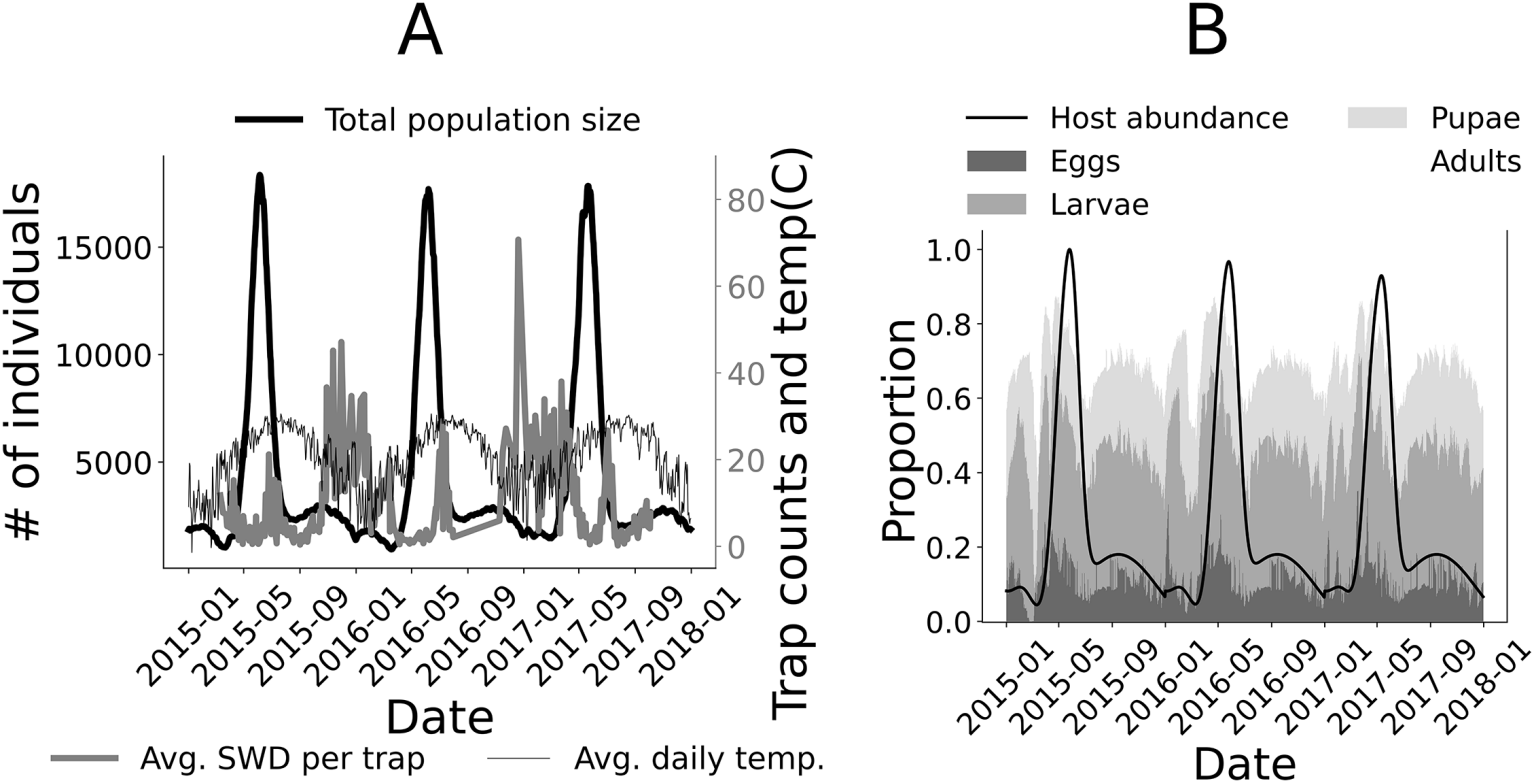
Testing the effect of host availability. Fecundity modifications by temperature were removed to assess the impact of host availability on population dynamics. A) Population density compared to trap catch data, 2015-2017. Simulated population size on left y-axis; trap counts and temperature on right y-axis. B) Proportion of life stages in the simulated population during the period of trap catch data; cumulative proportion of the population for each life stage and host availability on left y-axis; temperature on right y-axis. ‘Repro’ = reproductive; ‘temp’ = temperature.

**Fig 5:**
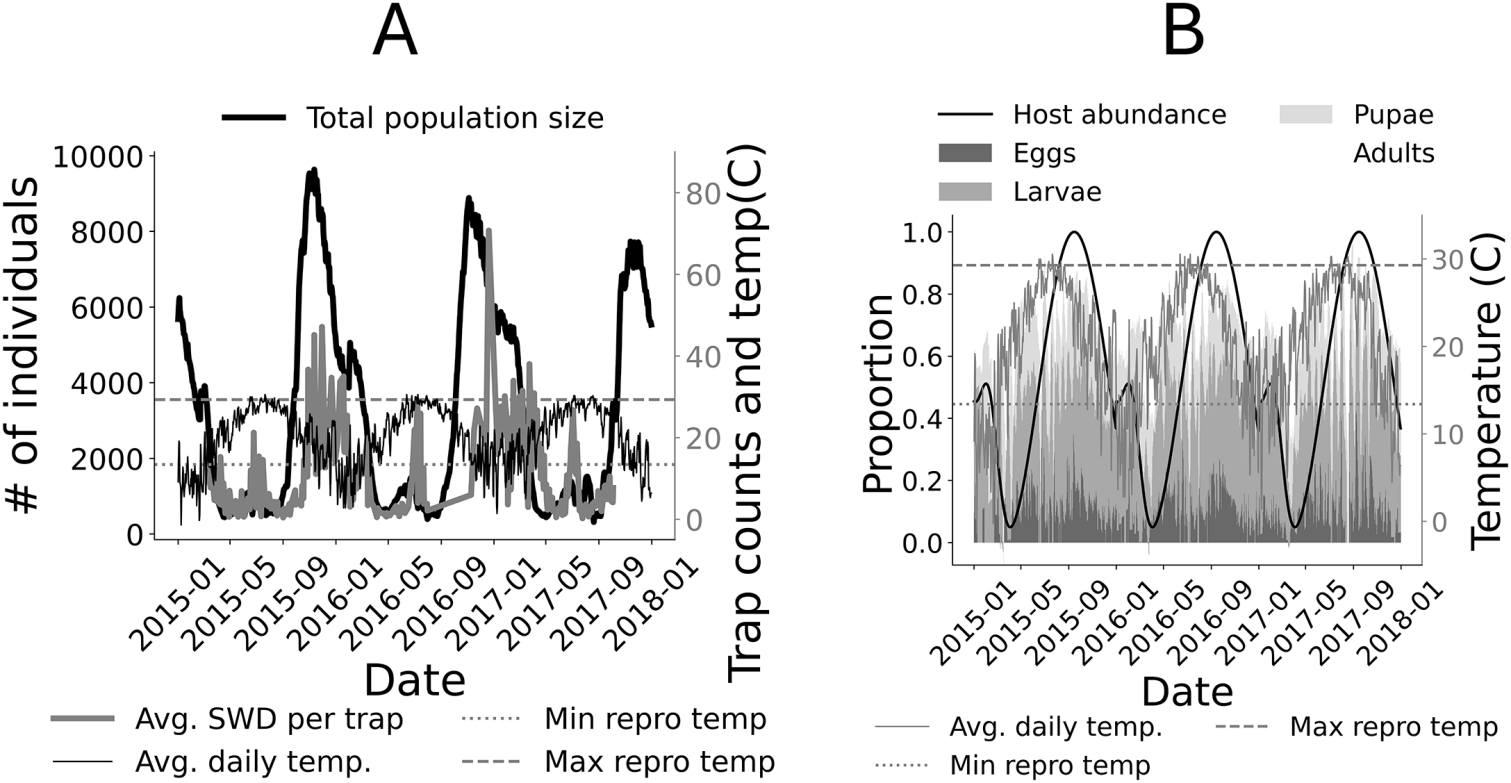
Population dynamics with only non-crop hosts. Crop hosts were removed from the simulation so that only non-crop hosts were available to SWD. A) Population density compared to trap catch data, 2015-2017. Simulated population size on left y-axis; trap counts and temperature on right y-axis. B) Proportion of life stages in the simulated population during the period of trap catch data; cumulative proportion of the population for each life stage and host availability on left y-axis; temperature on right y-axis. ‘Repro’ = reproductive; ‘temp’ = temperature.

**Fig 6:**
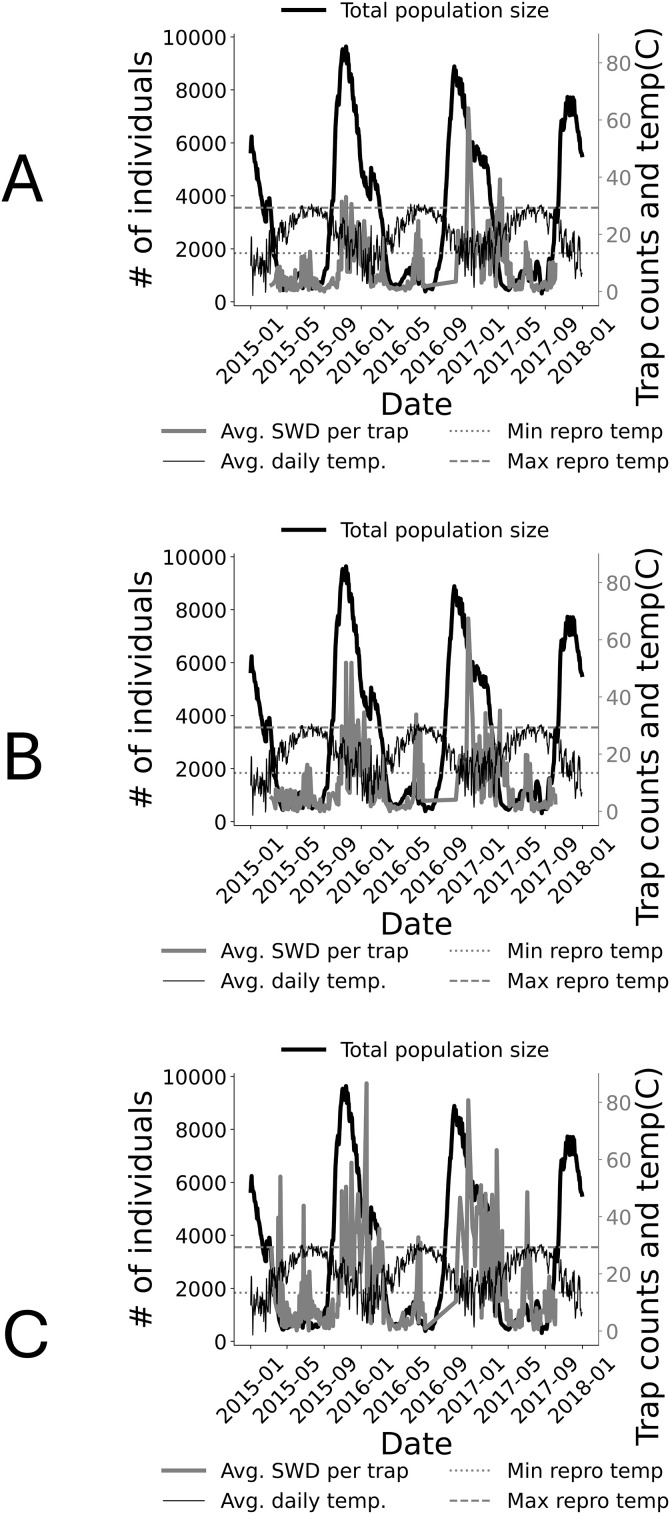
Comparing non-crop host simulation results to trap catch data in each type of habitat. Crop hosts were removed from the simulation so that only non-crop hosts were available to SWD. Results were compared to each of the three trap habitats: crop (blueberry) field (A), border between the crop field and unmanaged area (e.g., woods) (B), and in the unmanaged area (C). Simulated population size on left y-axis, trap counts and temperature on right y-axis for all plots. ‘Repro’ = reproductive; ‘temp’ = temperature.

**Fig 7:**
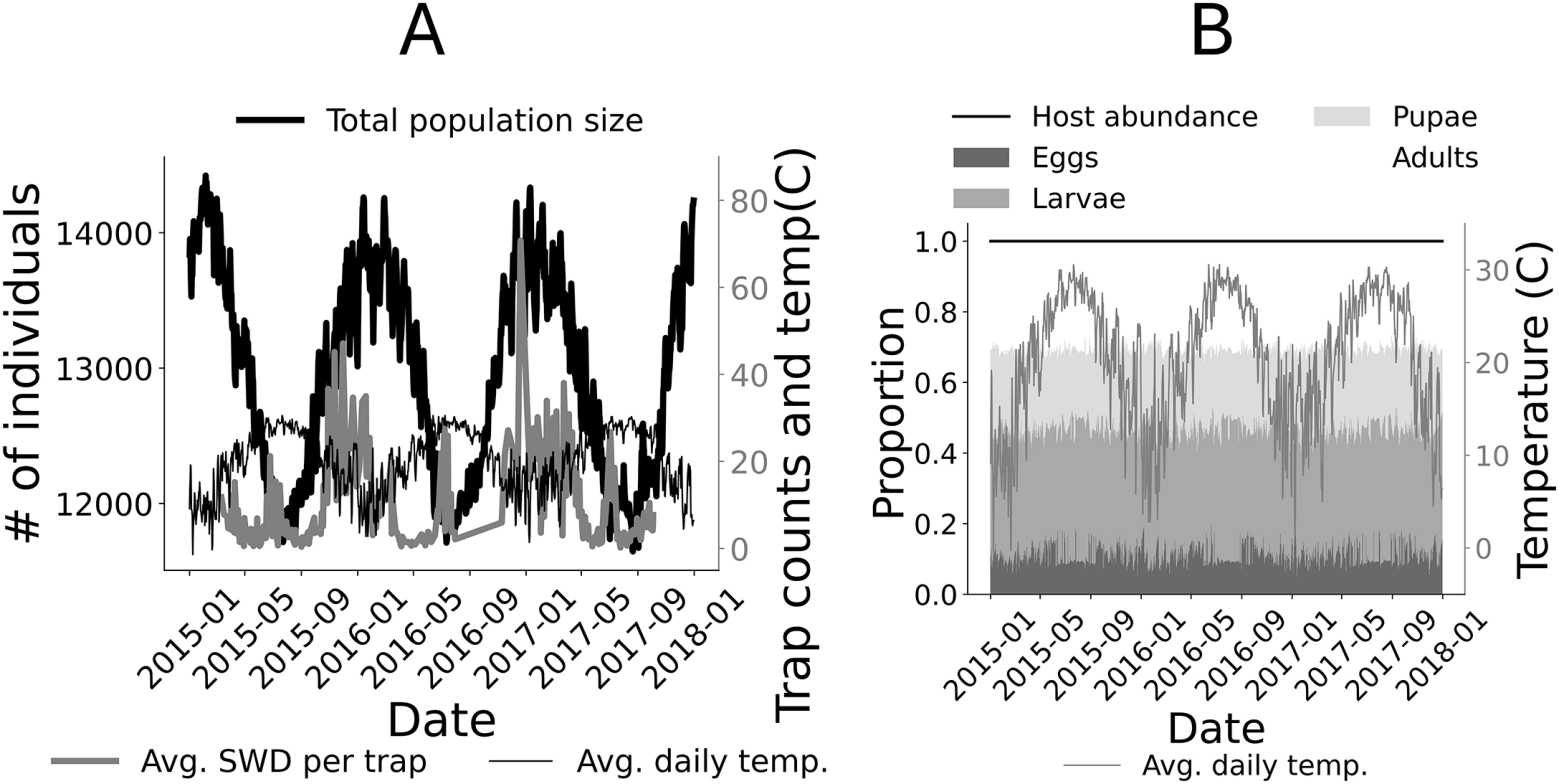
Control simulation. Fecundity modifications by both host availability and temperature were removed as a negative control simulation. A) Population density compared to trap catch data, 2015-2017. Simulated population size on left y-axis; trap counts and temperature on right y-axis. B) Proportion of life stages in the simulated population during the period of trap catch data; cumulative proportion of the population for each life stage and host availability on left y-axis; temperature on right y-axis. ‘Temp’ = temperature.

Fecundity of female SWD was modified by the population density of immatures in all simulations. Fecundity of female SWD was also modified by temperature and host availability under the standard simulation conditions (Fig 2). Host availability (Fig 3) or temperature (Fig 4) modifications to fecundity were removed from the simulation when assessing the impact of the alternate factor on the population dynamics of SWD. Fecundity modifications by host availability were removed by setting the total abundance of hosts to 1 throughout the duration of the simulation. Temperature modifications to fecundity were removed by setting the temperature modification factor to 1 and allowing reproduction at any temperature. Additionally, crop hosts were removed from the simulation and the non-crop host distribution was scaled up to 1, from 0.3, to assess the importance of non-crop hosts (Fig 5). The simulation results without crop hosts were also compared against the trap catch data for each of the three trap habitats individually, crop field, in the border between the crop field and the unmanaged habitat, and the unmanaged habitat, to assess the importance of trap habitat on the fit to the results (Fig 6). Fecundity modifications by both host availability and temperature were removed to serve as a negative control simulation (Fig 7). See Tables 1 and 2 for a list of functions and parameter values used in the simulation.

**Table 1:** Functions and descriptions of variables used in simulation.

Function	Use	Variable	Base values	Reference
**Gaussian:**	• Crop host availability	*a* = amplitude *µ* = center of distribution *σ* = standard deviation *x* = day of year	*S. highbush* *a* = 1 *µ* = DD^†^ dependent *σ* = 21.00 *Rabbiteye* *a* = 1 *µ* = DD^†^ dependent *σ* = 17.63	NA
$f(x)=ae^{-\frac{(x-\mu {)}^{\text{2}}}{\text{2}\times \sigma^{\text{2}}}}$	• Early non-crop host availability	*a* = amplitude *µ* = center of distribution *σ* = standard deviation *x* = day of year	*a* = 0.4 *µ* = 40 *σ* = 23	NA
	• Temperature-driven fecundity validation	*a* = amplitude *µ* = center of distribution *σ* = standard deviation *x* = avg. daily temperature	*a* = 19.8 *µ* = 21.0 *σ* = 5	NA
**Gompertz:** $f(x)=e^{\left(-\frac{b}{a}{(e}^{\left(ax\right)}-\text{1})\right)}$	• Survival based on physiological age	*a* = shape parameter *b* = rate parameter *x* = physiological age (in degree days)	*a* = 0.005304 *b* = 0.001503	[20,32]
**Cauchy:** $f(x)=c\frac{\text{1}}{\pi }\Upsilon \left(\frac{\Upsilon^{\text{2}}}{x-x\text{0}}+\Upsilon^{\text{2}}\right)$	• Reproduction based on physiological age	*c* = constant *π* = 3.14… *x0* = center of distribution *ϒ* = scale parameter *x* = physiological age (in degree days)	*c* = 2,120.576 *π* = 3.14… *x0* = 410 *ϒ* = 45	[20]
**Sine wave:** $f(x)=c+a\text{sin}\left(\text{2}\pi \frac{x-x\text{0}}{hz}\right)$	• Degree day accumulation	*a* = amplitude *c* = constant *π* = 3.14… *x0* = center of wave *hz* = frequency of wave *x* = hour of the day	*a* = temp. dependent *c* = temp. dependent *π* = 3.14… *x0* = 12 *hz* = 24	[31]
	• Mid and late season non-crop host availability	*a* = amplitude *c* = constant *π* = 3.14… *x0* = center of wave *hz* = frequency of wave *x* = day of year	*a* = 0.5 *c* = 0.5 *π* = 3.14… *x0* = 167 *hz* = 365	
**Host-SWD Interaction:** $\frac{\alpha F}{\text{1}-\alpha F}$	Adjusts fecundity for host availability	*α* = ‘attack rate’ *F* = host availability	*α* = 2 *F* = sum of host avail. at a time step	[19]
**Density Dependence:** $\text{1}-\left(\frac{n}{kF}\right)$	Adjusts fecundity for immature population density	*n* = number of immature SWD *k* = ‘carrying capacity’ *F* = host availability	*n* = sum of immature SWD at a time step *k* = 10,000 *F* = sum of host avail. at a time step	[30]

^†^ DD = degree day.

NA = not applicable.

**Table 2:** Parameter values used in simulations.

			Value in simulation		
Parameter	Standard	Host Restraints Removed	Reproductive Temp. Restraints Removed	Crop Hosts Removed	Host and Temp. Restraints Removed
SWD DD min. (°C)^*^	7.2	7.2	7.2	7.2	7.2
SWD DD max (°C)^*^	30	30	30	30	30
Physiological age for eggs^†^	0 to 22.27	0 to 22.27	0 to 22.27	0 to 22.27	0 to 22.27
Physiological age for larvae^†^	20.28 to 118	20.28 to 118	20.28 to 118	20.28 to 118	20.28 to 118
Physiological age for pupae^†^	118.1 to 199.9	118.1 to 199.9	118.1 to 199.9	118.1 to 199.9	118.1 to 199.9
Physiological age for adults^†^	200–610	200–610	200–610	200–610	200–610
Physiological age for reproductive adults^†^	210–610	210–610	210–610	210–610	210–610
Max SWD reproductive temp (°C)	29.3	29.3	Na^§^	29.3	Na^‡§^
Min SWD reproductive temp (°C)	13.4	13.4	Na^§^	13.4	Na^‡§^
Blueberry DD base (°C)^†^	10	10	10	10	10
Crop host scalar	1.0	Na^‡^	1.0	0.0	Na^‡^
Non-crop host scalar	0.3	Na^‡^	0.3	1.0	Na^‡^

* Temperature threshold for calculating degree days (DD).

^†^ Physiological age as measured in accumulated DDs; death occurs at physiological age of 610 DDs (20).

^‡^ When host restraints were removed, distributions were set to a constant of 1, and host modifications to reproduction did not apply.

^§^ When temperature restraints were removed, the maximum physiological reproductive potential was only modified by host availability and density-dependent factors.

DD = degree days.

### Statistical analysis

The simulation results were tested for fit to the trap catch data using a Kolmogorov-Smirnov (KS) test (PROC NPAR1WAY with edf option) (Table 3). The KS test uses proportional data to evaluate if two datasets originate from the same distribution, and a significant result indicates that the two datasets do not likely originate from the same distribution. Data for simulation results were only included in the tests where the date matched the trap catch data. Data for simulation results and trap catch were transformed to proportion of the range for each dataset: y’_t_ = (y_t_ – y_min_) / (y_max_ – y_min_), where y’_t_ is the proportion of the range of the population size at time ‘t’, y_t_ is the population size at time ‘t’, and y_min_ and y_max_ are the minimum and maximum population sizes, respectively, for any given simulation or dataset. For example, if y_min_ = 200, y_max_ = 1000, and y_t_ = 500, then y’_t_ = (500–200) / (1000–200) = 0.375. Simulations in which restrictions to reproduction were removed were not tested for fit to the trap catch data because such simulations were used to assess the impact of temperature and host availability on reproduction, and not for fit to trap data. Each simulation that was tested was divided into 9 bins, corresponding to the x-axis date markers for Figs 2–7, to prevent artificially significant results due to the large number of observations in each simulation and dataset pair (c.a. 250); the KS test was run on the transformed data within each bin; the resulting number of observations per bin (i.e., observation pairs per KS test) ranged from 5 to 46. Results for each KS test were adjusted with the Holm-Bonferroni (HB) method for 9 tests within each simulation using the PROC MULTTEST statement and the ‘holm’ option. We use the number of non-significant bins per simulation (HB adjusted P-value > 0.05) to assess the match of different simulations to the trap catch data: simulations with more non-significant bins, or fewer significant bins, are a better match to the trap catch data (Table 3).

**Table 3:** Results of Kolmogorov-Smirnov test between simulation results and observed field data from 2015-2017^†^.

	Bin 1	Bin 2	Bin 3	Bin 4	Bin 5	Bin 6	Bin 7	Bin 8	Bin 9	
Dates	1/1/2015–4/30/2015	5/1/2025–8/31/2025	9/1/2015 to 12/31/2015	1/1/2016–4/30/2016	5/1/2016–8/31/2016	9/1/2016 to 12/31/2016	1/1/2017–4/30/2017	5/1/2017–8/31/2017	89/1/2017 to 12/31/2017	# of NS bins per simulation
**Simulation**										
**Standard** ^**‡**^	21 0.238 *0.591*	40 0.600 *<0.0001*	34 0.588 *0.0001*	31 0.484 *0.004*	23 0.608 *0.002*	5 1.000 *0.027*	46 0.674 *<0.0001*	43 0.465 *0.001*	11 1.000 *0.0002*	1/9
**Only non-crop hosts** ^**§**^	-------------	-------------	---------------	-------------	-------------	---------------	-------------	-------------	---------------	---------------
**All habitats** ^**§**^	21 0.429 *0.211*	40 0.300 *0.219*	34 0.647 *<0.0001*	31 0.484 *0.011*	23 0.261 *0.829*	5 0.800 *0.245*	46 0.391 *0.012*	43 0.186 *0.829*	11 0.636 *0.140*	6/9
**Crop habitat** ^**§**^	21 0.429 *0.254*	40 0.250 *0.492*	34 0.676 *<0.0001*	31 0.581 *0.001*	23 0.304 *0.492*	5 0.800 *0.379*	46 0.391 *0.012*	43 0.209 *0.492*	11 0.545 *0.379*	6/9
**Border habitat** ^**§**^	21 0.429 *0.169*	40 0.350 *0.089*	34 0.618 *<0.0001*	31 0.484 *0.011*	23 0.261 *0.414*	5 0.800 *0.245*	46 0.370 *0.026*	43 0.233 *0.391*	11 0.636 *0.116*	6/9
**Unmanaged habitat** ^**§**^	13 0.385 *1.000*	37 0.351 *0.154*	34 0.618 *<0.0001*	31 0.387 *0.154*	23 0.174 *1.000*	5 0.800 *0.489*	46 0.239 *0.720*	43 0.209 *1.000*	11 0.364 *1.000*	8/9
**# of NS**^**¶**^ **simulations per bin**	5/5	4/5	0/5	1/5	4/5	4/5	1/5	4/5	4/5	---------------

^†^ The results of the Kolmogorov-Smirnov (KS) test for each bin in each simulation are listed in the following order: the number of sample dates per bin is listed first, the KS test statistic (D) is listed below the number of samples, and the P-value (after a Holm-Bonferroni adjustment for 9 test within each simulation) is listed in *italics* below the test statistic.

^‡^ The standard simulation included crop and non-crop hosts, scaled to 1 and 0.3, respectively, and was tested against the average (by date) trap catch data for all habitat types.

^§^ Simulations included only non-crop hosts, scaled to 1 from 0.3, and were tested against the average (by date) trap catch data from the indicated habitat type.

^¶^ NS = non-significant.

## Results

Days to peak fruit set (50% blue) for southern highbush and rabbiteye were 116 and 144 in 2023, and 119 and 154 in 2024, respectively, corresponding to average degree days to peak fruit for southern highbush and rabbiteye varieties of 697.66 and 1087.80, respectively. The duration of fruiting was 84 and 70.5 days, for southern highbush and rabbiteye varieties, respectively. Crop hosts were available for a short period of time and availability peaked during late spring (Fig 2).

Eighteen potential non-crop host species were identified in habitat adjacent to blueberry fields in southern Georgia throughout 2015 [7]. The data for number of fruiting non-crop host species was visually fit with a normal Gaussian function (*a* = 0.4, *µ* = 40, *σ* = 23) added to a sine wave (*c* = 0.5, *a* = 0.5, *x0* = 167, *hz* = 365) as a measure of the relative abundance of fruiting non-crop host availability throughout the year (Table 1). The composite function provided a good fit to the observed data (t-value = 12.75, df = 1, P-value < 0.0001), produced an adjusted r^2^ value = 0.936 in the linear regression and a regression coefficient that was not significantly different from 1 (regression coefficient  = 0.933, F-value = 0.85, df = 1, 10, P-value = 0.379). Non-crop hosts were available throughout most of the year but were minimally available during the spring and early summer, and were most abundant in late autumn (Fig 5).

The parameter combination of *µ* = 21 and *σ* = 5 in the Gaussian equation for temperature driven fecundity produced the lowest MSE (0.27), predicted the observed egg numbers well (t-value = 35.73, df = 1, P-value < 0.0001), produced an adjusted r^2^ value = 0.996 in the linear regression and a regression coefficient that was not significantly different from 1 (regression coefficient = 1.08, F-value = 7.23, df = 1, 3, P-value = 0.075) (Table 1).

The arbitrary choice of starting population size and age had a negligeable impact on the simulation results beyond 2014, indicating that the results during the period of trap catch data were not impacted by the size and age of the cohorts in the starting population (S4 Fig). The simulation showed seasonal variation in SWD population dynamics: population density peaked in late spring and declined immediately afterwards in standard simulation conditions (Fig 2A). The timing of rapid population decline in late spring matched reasonably well with the drop in trap catches during this period. However, the standard simulation did not predict the rise in trap catches seen during autumn and winter (Fig 2A). Population decline in the simulation was associated with increased proportions of adults and decreased proportions of immatures during periods of high summer temperatures and declining host availability (Fig 2B). The standard simulation only produced one bin that was not significantly different from the trap catch data, out of nine total bins (Table 3).

When reproductive constraints by host availability were removed, the population size was approximately stable when temperatures were within the reproductive range of SWD (Fig 3A). The proportion of adults increased, and the proportion of immatures decreased during periods of high summer temperatures, and to a lesser extent during extreme cool winter temperatures, when host availability was constant at 1 throughout the simulation (Fig 3B). The simulation results did not match the trap catch data, except perhaps for the timing of population decline in late spring (Fig 3B).

Similar to the standard simulation, population density peaked in late spring during blueberry season and immediately crashed thereafter when temperature modifications to reproduction were removed (Fig 4A). The results predicted the timing of trap catch decline in the early summer as well as the timing of trap catch increase in autumn, but did not match the relative amplitude changes of the trap data (Fig 4A). The proportion of adults increased, and the proportion of immatures decreased during periods of low or declining host availability (Fig 4B).

The simulation results matched the trap catch data very well during all seasons when crop-hosts were completely removed from the simulation, leaving only non-crop hosts available throughout the year; additionally, the proportion of adults increased and the proportion of immatures decreased during periods where temperatures were near or exceeded the reproductive temperature thresholds of SWD, even when host availability was high or increasing (Fig 5). The pattern of population dynamics matched the trap catch data from all trap habitats, although there was a trend of increased trap catches from crop field to border to unmanaged habitat (Fig 6). The simulation with only non-crop hosts in the landscape produced 6 bins that were not significantly different from the trap catch data from all trap habitat types, out of nine total bins (Table 3). The simulation produced six non-significant bins when compared to trap catch data from either the crop field or border habitats (Table 3). The simulation produced eight non-significant bins when results were compared to trap catch data from the surrounding unmanaged habitat (Table 3).

The population fluctuated around 13,000 individuals, showing stable proportions of life stages across time, when both temperature and host constraints to reproduction were removed. These results indicate that the impacts of temperature and host availability in the modified simulations were not artifacts of the simulations (Fig 7)

## Discussion

This simulation used functions for survival and reproduction, based on physiological age, to simulate SWD population dynamics using host availability (crop and non-crop hosts) and temperature data from southern Georgia (20). We compared the results of the simulation to trap catch data collected from 2015 to 2017 at seven blueberry farms in southern Georgia. The simulation showed that the SWD population peaked in spring and was followed by rapid decline, matching the decline in early summer trap catches, but failed to predict the increase in trap catches during autumn and winter under standard simulation conditions (Fig 2). When host and temperature restraints on reproduction were removed, the simulation showed that high summer temperatures (and cooler winter temperatures, to some degree) and lack of host availability, respectively, constrained SWD reproduction, causing population declines (Figs 3 and 4). The simulation results reflected the seasonal trends in trap catch data when crop hosts were removed from the simulation, leaving only non-crop hosts available for SWD reproduction; the closest match occurred when the simulation results were compared to trap catch data from the unmanaged habitat (Table 3). These results provide insights to the population ecology of SWD that can be used to improve management of this pest in southern Georgia, USA.

Temperatures outside the reproductive range of SWD constrained reproduction, as expected (Figs 2, 3, 5) [23,26,27]. The daily average temperature during the summer in southern Georgia, USA, frequently exceeded the maximum reproductive temperature thresholds of SWD; to some extent the daily average temperature during the winter falls below the minimum reproductive temperature of SWD (Figs 2, 3, 5) [20,23]. The simulated SWD population declined during these periods and showed increased proportions of adults and decreased proportions of immatures, indicating constrained reproduction. This was observed even when constraints of host availability on reproduction were removed (Fig 3B). SWD populations are expected to decline during the summer in other locations with extended periods of above the maximum reproductive temperature, such as in Wilmington, North Carolina, USA, and Parlier, California, USA [21,33]. Extended periods of temperatures outside the reproductive threshold of SWD are expected to constrain SWD reproduction and cause population decline, regardless of host availability.

Behavioral and physiological adaptations allow SWD to persist through seasonal changes and periods of unfavorable environmental conditions. Phenotypic traits (i.e., pigmentation of the cuticle, cold tolerance, wing morphology, etc.) shift according to thermal reaction norms and improve fitness in response to changes in environmental conditions, similar to the adaptive tracking described in *D. melanogaster* [11,34,35]. Adult SWD limit their activity to crepuscular periods and utilize microhabitats with more favorable conditions to persist through extreme environmental conditions [36,37]. Including these microclimate data and use patterns by pests can improve the power of predictive tools [38]. Additionally, the reported temperature metrics for life history events demonstrate variability among populations [27]. For example, the reported maximum reproductive temperature of the population studied in Evans et al. (2018), collected in Georgia, USA, was 30°C while the population studied in Tochen et al (2014), collected in Oregon, USA, was unable to reproduce at 30°C. Differences in methods and analyses are expected to produce variation in results, but geographically distinct populations of SWD that are uniquely adapted to their local conditions could be another source of variation [27]. To improve model simulations and other management tools, more research is needed to understand how SWD populations are adapted to local conditions and how they respond to seasonal changes [13].

Periods of low or declining host availability constrained SWD reproduction (Figs 2, 4). The peaks and valleys of the proportions of adults and immatures followed the pattern of host availability when temperature constraints on reproduction were removed, reinforcing the interpretation that host availability is a major factor affecting SWD reproduction (Fig 4B). Pfab et al. (2018) also showed that the peak population density in a simulated population of SWD tracked shifts in the timing of peak host availability. These observations support the greater context of resource limitation, or density-dependent feedback, for SWD populations. Competition for resources reduces fecundity and survival of SWD and increase the chances of larvae pupating outside the host substrate, which increases the risk of desiccation, parasitism, and predation [33,39–41]. Taken together, these results suggest that SWD reproduction is constrained, and additional aspects of SWD biology and ecology need to be explored which in turn will impact existing management actions, when host resources are limited or are in decline.

The results of the simulation approximated the trap catch data year-round when only non-crop hosts were available to SWD (Fig 5). These conditions also produced more non-significant bins, compared to the standard simulation, in the KS test (Table 3). This pattern was evident regardless of the trap habitat (i.e., crop field, border between crop field and unmanaged habitat, and unmanaged habitat), and comparison to trap catch data from the unmanaged habitat produced the most non-significant bins (Table 3, Fig 6). Drummond et al. (2019) captured SWD as early as one day after an insecticide treatment in wild blueberry fields, supporting the hypothesis that SWD in crop fields emigrate from adjacent unmanaged habitat [42]. Furthermore, the trend of increased trap catches of SWD from crop field to border to unmanaged habitat suggests a distance component to the relationship between habitat type and trap catches (Fig 6) [42]. The blueberry fields in which the trap data were collected were commercial fields where growers employed typical pest management and harvest practices. This may help explain why the trap catches were lower than expected during the blueberry season, and why the simulation results in these scenarios matched the trap catch data: SWD populations were suppressed with insecticides and crop host resources were continually removed, creating a relatively less favorable habitat for SWD and few resources in the crop field [43]. The simulation results from January to May produced a poor match to the trap catch data in both 2016 and 2017, except when compared to the data from the unmanaged habitat, further supporting the conclusion that unmanaged habitat surrounding crop fields, and the associated non-crop hosts, play an important role in population dynamics of SWD (Table 3). Grant and Sial (2021) concluded that non-crop hosts are important to sustain SWD populations when blueberries are not available [7]. Despite the broad interpretation possibilities of trap catch data, these results highlight the importance of host availability and imply that non-crop hosts are an important resource for SWD, impacting population dynamics in crop fields year round [44,45].

Host quality can affect the reproductive capacity of SWD [8,33]. Non-crop host species in southern Georgia vary in quality as a host resource and did not produce as many adult SWD as blueberry, which were preferred by SWD if given the choice [7]. Lower quality resources increase intraspecific competition, which has the potential to alter the dynamics and structure of a population [1,46–49]. Scaling the relative abundance of non-crop host availability to 0.3 in the standard simulation could also be interpreted as non-crop hosts are a lower-quality resource for SWD compared to crop hosts (Figs 2, 4). The lack of many non-significant bins under the standard simulation conditions suggests that there may not be a significant difference in quality between crop and non-crop hosts in a field setting, or that it is incorrect to interpret the scaling factor as a measure of host quality (Table 3, Fig 2). However, the larger number of non-significant bins in the simulations with only non-crop hosts suggests that non-crop hosts are of sufficient quality to impact SWD population dynamics, even in adjacent crop habitat (Table 3, Figs 5–6). The ability of non-crop hosts to sustain SWD populations is not well studied but would be valuable information to potentially disrupt the invasion ecology of SWD into crop habitats [47].

The simulation produced no non-significant bins across all habitat categories of trap catch data for the period of Sept.1, 2015 to Dec. 31, 2015 (Table 3: Bin 3). The simulation also produced poor matches to the trap data for the periods of Jan.1 to March 30 in 2016 and 2017, except when comparing the results to the trap catch data from the unmanaged habitat (Table 3: Bins 4 and 7). These results indicate that the current simulation may be limited to track the SWD trap catch data in certain instances. Further refinement will be needed to more accurately capture SWD population dynamics during these periods when patterns of temperature and host availability were fluctuating. Despite these specific limitations, our results support the current literature that temperature is a major factor affecting SWD reproduction, and clearly indicate the importance of host availability, non-crop hosts, and unmanaged habitat for SWD population dynamics (Table 3, Figs 5–6).

This simulation addressed a gap in the predictive tools for SWD in blueberries in southern Georgia, USA. The standard simulation conditions predicted the drop in trap catches at the end of the blueberry season but did not match the increase in trap catches during the autumn and winter (Fig 2). The results agree with other reports that high summer temperatures constrain SWD reproduction and lead to population decline [21,33] (Fig 3). The simulation also predicted that periods of low or declining host availability constrain SWD reproduction, and suggested that the population structure (i.e., proportions of adults and immatures, including eggs, larvae, and pupae) tracked patterns of relative host abundance through the season (Fig 4). The simulated population density matched the trap catch data across all seasons of the year, when non-crop hosts were the only resource available to the simulated SWD population; this indicates that non-crop hosts play an important role in the population dynamics of SWD (Table 3, Figs 5–6). These results contribute to the current literature that show that temperature, host availability, and resource quality are important aspects for SWD population dynamics [7,39,45,47]. Determining how geographically distinct populations of SWD respond to localized temperature and seasonal changes will improve predictive simulations, and future research that describes the quality and suitability of non-crop hosts to support SWD populations is particularly warranted. Answering these questions will identify new aspects of SWD biology and ecology that may be targeted to improve sustainable management of this insect pest.

## Supporting information

S1 Fig Sensitivity analysis for initial population size and age. Population dynamics of 10 initial cohorts of ages equally dispersed across the physiological age range for SWD adults (210–610 accumulated degree days), with variation of cohort size, ranging from 10 to 1280 adult individuals, doubling at each increment (A), and of an initial population comprised of a single cohort of 500 adult SWD at different physiological ages ranging from 210 to 600 accumulated degree days (B).
(TIF)

S2 Fig Sensitivity analysis for carrying capacity. Population dynamics with variations of carrying capacity, from 6000 to 14000, in increments of 2000: for crop and non-crop hosts (1:0.3) (A), and for non-crop hosts only (B).
(TIF)

S3 Fig Sensitivity analysis for proportions of crop and non-crop hosts in the landscape. Population dynamics when varying the scalar of non-crop hosts: for a crop host scalar of 1 (A), and when crop hosts were removed completely, leaving only non-crop hosts (B).
(TIF)

S4 Fig Sensitivity analysis for crop host phenology. Host availability (A) and SWD population dynamics (B) when crop host phenology was varied; Metrics for 50% blue fruit and fruit duration were varied using values from 2023, the average of 2023 and 2024, and 2024. The timeframe of the graphs was reduced to the three years of the trap catch data (2015–2018), from the full five and a half years, to improve resolution of differences.
(TIF)
