# Supplementary figures and images for: Using a simulation model to assess the significance of temperature and host availability for population dynamics of Drosophila suzukii (Diptera: Drosophilidae)
Source: PLoS One. 2026 Jul 15;21(7):e0351723. doi: 10.1371/journal.pone.0351723 (PMC13372139; doi:10.1371/journal.pone.0351723)

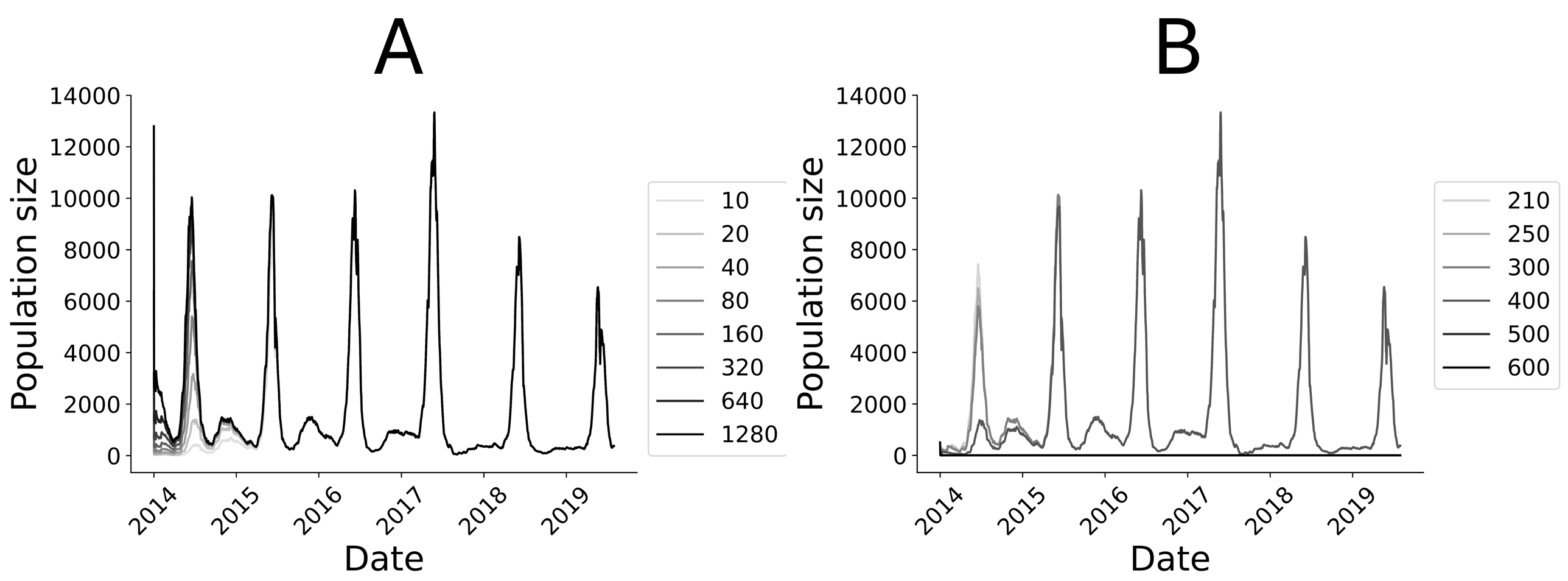

Supplement: S1 Fig — Population dynamics of 10 initial cohorts of ages equally dispersed across the physiological age range for SWD adults (210–610 accumulated degree days), with variation of cohort size, ranging from 10 to 1280 adult individuals, doubling at each increment (A), and of an initial population comprised of a single cohort of 500 adult SWD at different physiological ages ranging from 210 to 600 accumulated degree days (B). (TIF) [file pone.0351723.s001.tif]

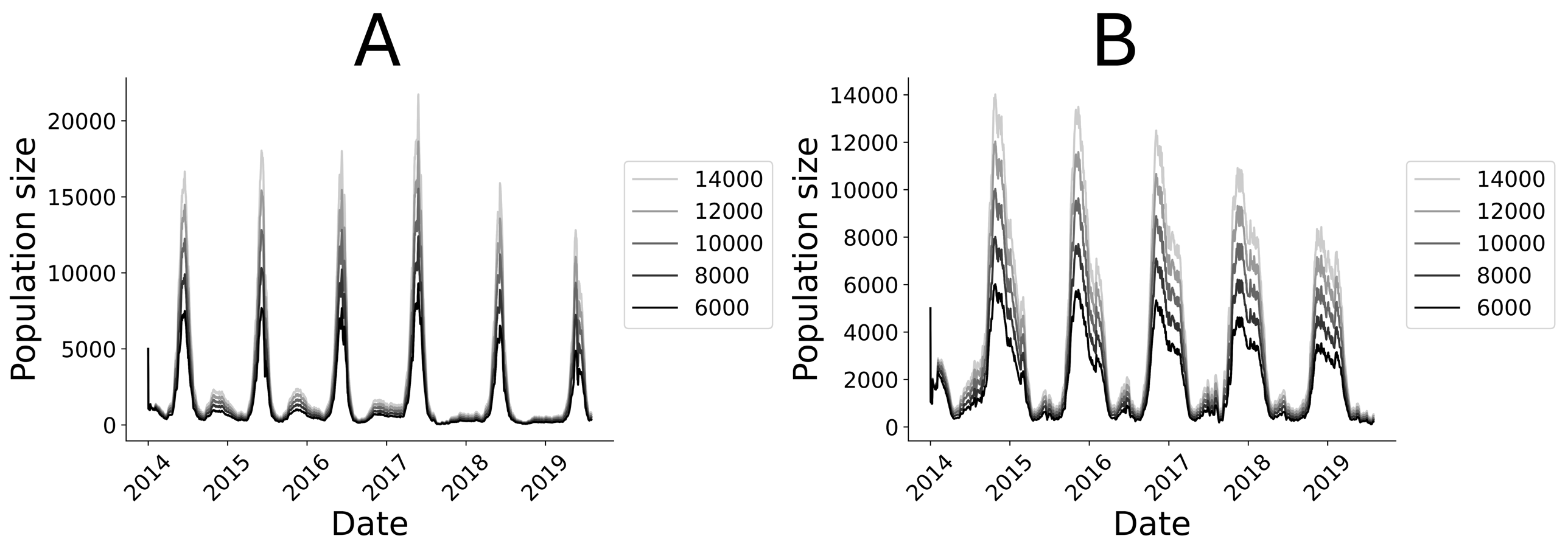

Supplement: S2 Fig — Population dynamics with variations of carrying capacity, from 6000 to 14000, in increments of 2000: for crop and non-crop hosts (1:0.3) (A), and for non-crop hosts only (B). (TIF) [file pone.0351723.s002.tif]

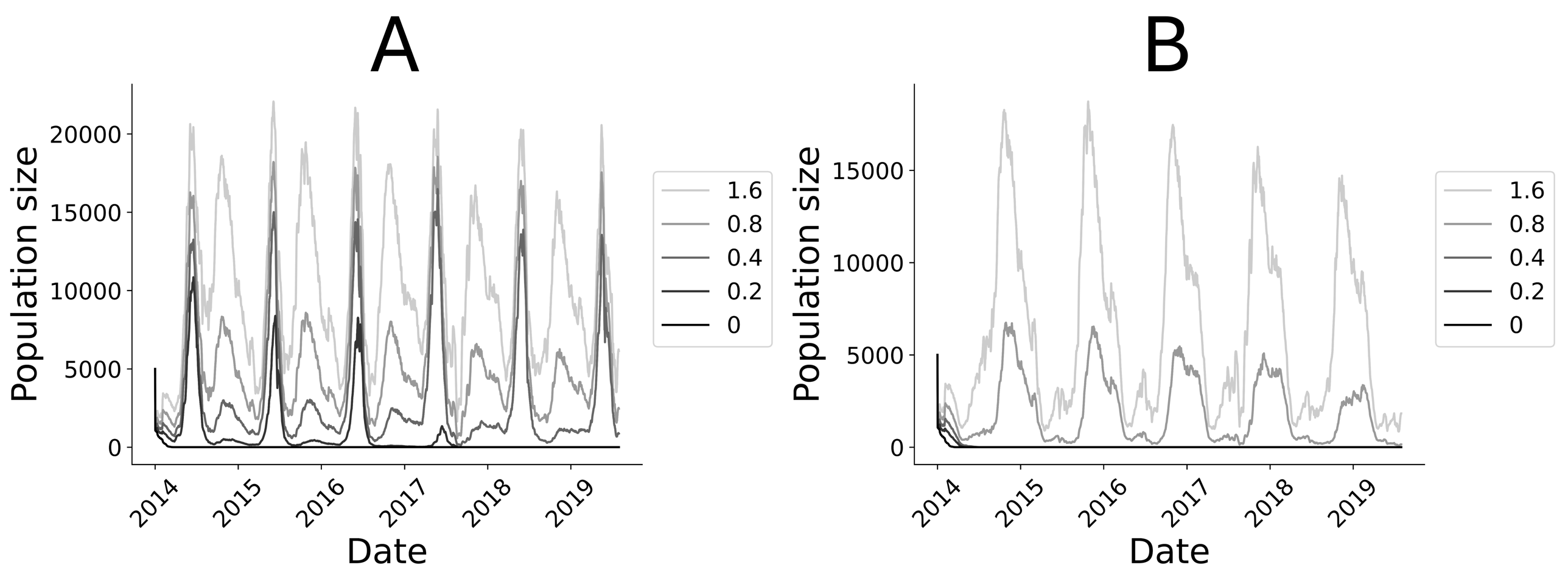

Supplement: S3 Fig — Population dynamics when varying the scalar of non-crop hosts: for a crop host scalar of 1 (A), and when crop hosts were removed completely, leaving only non-crop hosts (B). (TIF) [file pone.0351723.s003.tif]

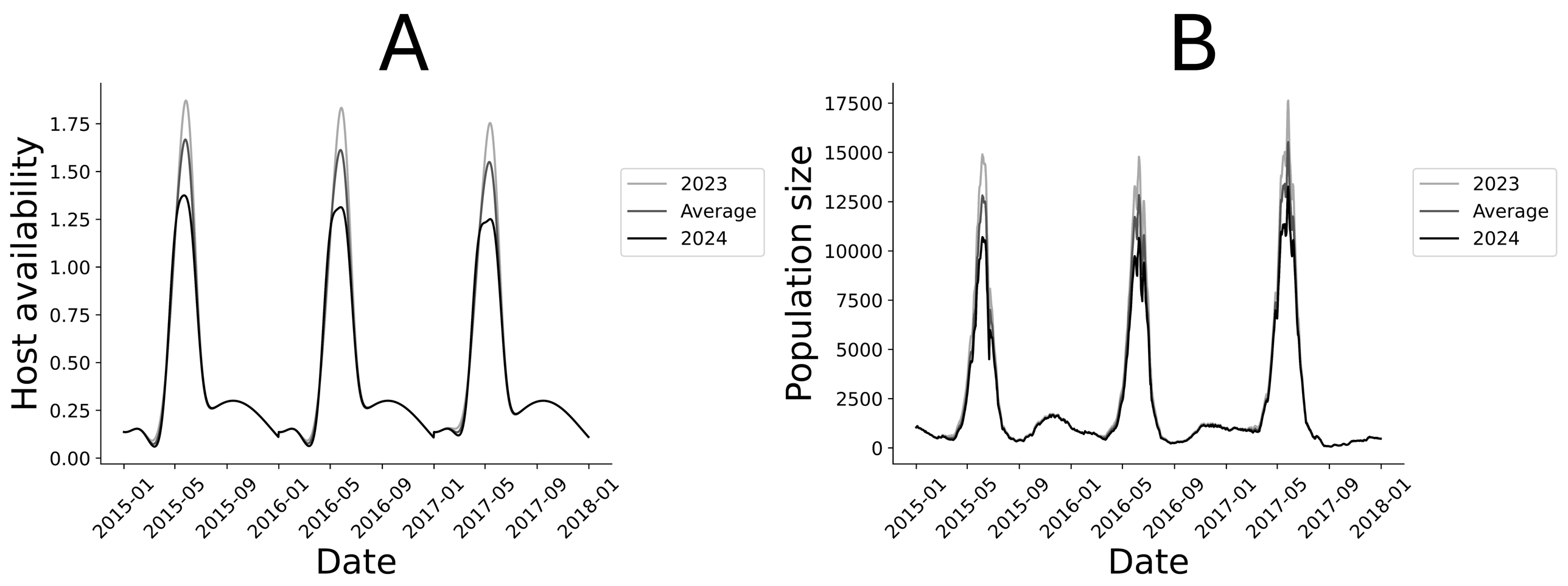

Supplement: S4 Fig — Host availability (A) and SWD population dynamics (B) when crop host phenology was varied; Metrics for 50% blue fruit and fruit duration were varied using values from 2023, the average of 2023 and 2024, and 2024. The timeframe of the graphs was reduced to the three years of the trap catch data (2015–2018), from the full five and a half years, to improve resolution of differences. (TIF) [file pone.0351723.s004.tif]
